# A systematic review of posterior reversible encephalopathy syndrome in pregnant women with severe preeclampsia and eclampsia

**DOI:** 10.1177/1753495X221150302

**Published:** 2023-01-17

**Authors:** Dalal A Tawati, Wee-Shian Chan

**Affiliations:** 1 Department of Obstetrics and Gynecology, 8166 University of British Columbia, Maternal Fetal Medicine Subspecialty Program, Children's & Women's Hospital, Vancouver, BC, Canada; 2 Department of Medicine, 8166 University of British Columbia, Vancouver, BC, Canada

**Keywords:** Leukoencephalopathy, neuroimaging, cerebral edema, hypertension, hypertensive disorders of pregnancy

## Abstract

**Background:**

The association of posterior reversible encephalopathy syndrome (PRES) and severe preeclampsia/eclampsia has been established but the frequency is uncertain.

**Objectives:**

To determine the frequency of PRES in severe preeclampsia or eclampsia.

**Methods:**

We searched published articles in PubMed, Cochrane library, Embase, and CINAHL from 1990 to 2020. We included articles that reported on six or more cases of PRES with eclampsia or severe preeclampsia who underwent neuroimaging during pregnancy or up to 6 weeks postpartum.

**Results:**

We identified 29 studies presenting data on 1519 women with eclampsia or severe preeclampsia. Among 342 women with eclampsia who had neuroimaging, 176 (51.4%) were diagnosed with PRES. Of 121 women with severe preeclampsia, 24 (19.8%) had PRES. The pooled maternal death rate was 5.3% (21/395).

**Conclusion:**

PRES is commonly reported on neuroimaging of women with eclampsia/ severe preeclampsia. The role of neuroimaging in eclampsia and especially in women with severe preeclampsia requires re-evaluation as further management is often dictated by this finding.

## Introduction

Preeclampsia affects 2–5% of pregnancies in North America, and up to 18% in developing countries.^1^ It is a leading cause of both maternal and fetal morbidity and mortality. The pathophysiology of preeclampsia involves widespread endothelium dysfunction, affecting multiorgan systems such as hepatic, hematological, renal, cardiovascular, and the central nervous system. Posterior reversible encephalopathy syndrome (PRES) is a radiological finding that was described by Hinchey et al. in 1996 .^2^ The authors described fifteen patients (three of whom had eclampsia) presenting with clinical symptoms (headache, altered consciousness, seizures, and vomiting) who underwent neuroimaging, which revealed edema involving the white matter in the posterior portions of the cerebral hemispheres, especially in the parieto-occipital regions.^2^ Although the term PRES was first used in the study, a prior observational study involving neurological imaging of eclamptic women described similar brain findings.^3^ In recent decades, as neuroimaging, particularly magnetic resonance imaging (MRI), became widely available, more studies describing the neuroimaging findings in these pregnant women have been performed. The finding of PRES in these women is recognized as a severe and serious complication of preeclampsia warranting delivery.^4^ Symptoms such as headache and blurred vision, which are frequent in women with preeclampsia are also frequently described in women with PRES.^2^ The need to image all preeclamptic women, especially when they present with neurological symptoms, to exclude PRES, is uncertain.

### Objective

We conducted this systematic review to describe the frequency of PRES in women presenting with preeclampsia/eclampsia and second to elucidate the features and presentation of PRES in these women.

## Methods

A review of the literature was conducted in accordance with PRISMA methodological guidelines (see Supplemental material). The two authors (D.T and WS.C) independently performed the literature systematic search, study selection, quality assessment, and data extraction. Disagreements about study inclusion or data extraction were resolved by discussion and consensus between the authors. The two authors received no funding in the preparation and processing of this systematic review.

### Search strategy and eligibility criteria

A systematic literature search was conducted in English on PubMed (MEDLINE), Embase, CINAHL, and Cochrane up to 20 April 2020, by the two authors, independently. The search strategy was developed using multiple terms that described the population (preeclamptic, pregnancy, and pregnant) combined with the outcome of brain edema or PRES as well as the intervention of brain imaging or MRI.

We included studies that met our prior agreed population of interest, intervention, comparison, outcomes, and study design (PICOS) criteria. Eligible studies included (a) women with eclampsia or preeclampsia who develop new-onset neurologic symptoms during pregnancy or 6 weeks after delivery, (b) use of neuroimaging MRI or computed tomography (CT) scan to confirm the diagnosis of PRES and/or described its features, and (c) the analysis only included studies written in English.

We excluded studies when they reported women who were known to have epilepsy and who had evident brain pathology such as tumors, space-occupying lesions, or brain cysts. Case reports and case series with less than 6 women were also excluded. For studies that appeared to have duplicate reporting of the same data over time, we included only the most recent or more comprehensive report.

### Study selection and data extraction

Records that were retrieved from our database search were all imported into Covidence 2019, a web-based collaboration software platform that streamlines the production of systematic and other literature reviews. All obtained citations were screened by titles and abstracts. Full texts of primary screened titles and abstracts were then reviewed and evaluated carefully for final approval of the studies included. All steps were performed independently by the two researchers (D.T. and WS.C.) and the evaluation was based on the inclusion criteria mentioned above.

Each included study was assigned a unique identification number, and data were extracted, collected, and entered on a database manager software spreadsheet by two independent researchers.

### Data synthesis

Abstracted data included: study design, country of study, and year of publication. We also collected information regarding our primary outcome: number of eclamptic/preeclamptic women diagnosed with normal neuroimaging and with a diagnosis of PRES, modality of neuroimaging, and whether or not all consecutive women were neuroimaged. Other variables of interest included signs and symptoms of PRES, affected areas of the brain, biochemical and hematologic findings, and maternal and fetal outcomes, including maternal mortality.

To determine our primary outcome, we pooled data from studies where consecutive women diagnosed with eclampsia and preeclampsia had neuroimaging and calculated the frequency of PRES observed in these women.

We pooled data from all eligible studies for the following secondary outcomes: (a) Areas of the brain most affected, (b) signs and symptoms frequently associated with eclamptic/ preeclamptic PRES, (c) reported maternal mortality, maternal intracranial hemorrhage frequencies, and (d) fetal outcomes including route of delivery and fetal APGAR score at birth.

## Results

**Figure 1.**
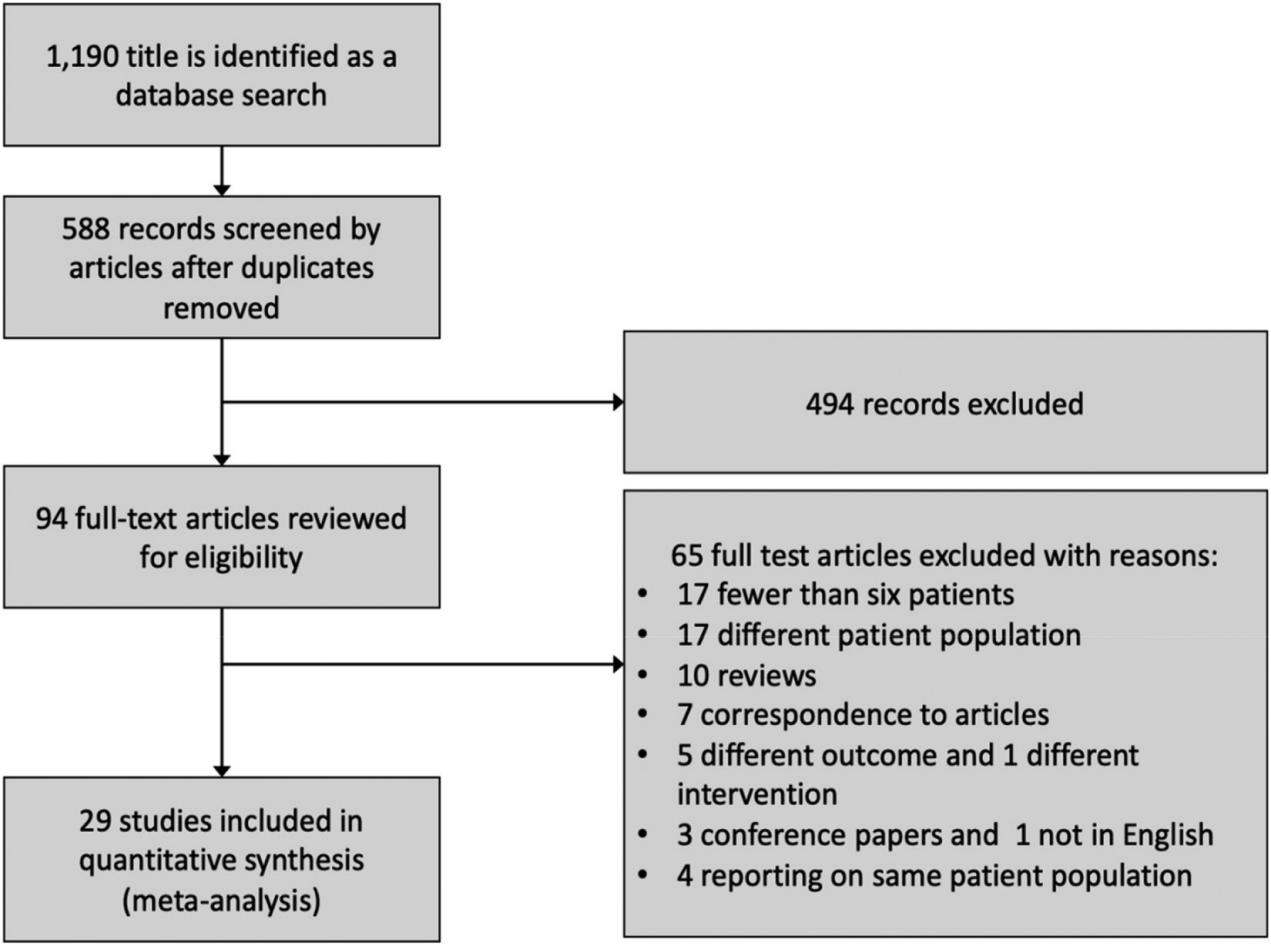
Study flowchart.

From our initial search, we identified 1190 citations. After primary screening of citations, we identified 334 articles that were potentially eligible for our analysis. On further screening, 240 articles were excluded on the basis of relevance and duplication. Two authors reviewed the remaining 94 articles, resulting in a final list of 29 articles in which the inclusion criteria were met (Figure 1). The 29 studies presented data on 1519 pregnant women with eclampsia/ severe preeclampsia who underwent neuroimaging studies.

Of the 29 included studies, 6 were prospective studies,^5–10^ the remainder were retrospective reviews.^11–33^ A summary of the characteristics of the included studies presenting data from 1519 women is presented in Table 1.

**Table 1.** Eligible studies included in the systematic review.

Study	Eclamptic, n	Preeclamptic, n	Confirmed PRES, n	Consecutive women imaged	Imaging modality	Frequency of PRES with eclampsia	Frequency of PRES with preeclampsia
Zeeman et al., 2004 (USA)^11^	27		25	No	MRI	92.59%	_
Striano et al., 2005 (Italy)^12^	7		7	No	CT/MRI	100%	_
Matsuda et al., 2005 (Japan)^5^	6	35	11	Yes	MRI	100%	14.28%
Topuz et al., 2007 (Turkey)^13^	58	62	54	No	CT/MRI	55.17%	35.48%
Shah et al., 2008 (USA)^14^	32		27	No	CT/MRI	77.5%	_
Mueller-Mang et al., 2009 (Austria)^15^	8	2	10	No	MRI	100%	100%
Roth and Ferbert, 2009 (Germany^16^	7		7	No	CT/ MRI	100%	_
Wagner et al., 2011 (USA)^17^	7		7	No	CT/ MRI	100%	_
Demir ret al., 2012 (Turkey)^18^	80	73	62	No	CT/ MRI	73.7%	4.1%
Liman et al., 2012 (Germany)^19^	15	9	24	No	MRI	100%	100%
Paul et al., 2013 (India)^20^	4	8	6	No	CT/ MRI	75%	37.5%
Brewer et al., 2013 (USA)^21^	47		46	No	CT/ MRI	97.87%	_
Junewar et al., 2014 (India)^22^	35		27	No	MRI	77.14%	_
Demirel et al., 2014 (Turkey)^23^	6	1	7	No	MRI	100%	100%
Marrone et al., 2014 (Brazil)^24^	9	9	18	No	MRI	100%	100%
Sen et al., 2014 (India)^25^	8	2	10	No	MRI	100%	100%
Kurdoglu et al., 2015 (Turkey)^6^	81		45	Yes	MRI	55.5%	_
Bembalgi et al., 2015 (India)^26^	11		11	Not clear	Not clear	100%	_
Hossain et al., 2015 (Pakistan)^27^	22		9	No	CT/MRI	40.9%	_
Mayama et al., 2016 (Japan)^7^	13	26	17	Yes	MRI	92.3%	19.2%
Fisher et al., 2016 (USA)^28^	8	38	9	No	MRI	62.5%	10.5%
Camara-Lemarroy et al., 2017 (Mexico)^29^	29		17	No	MRI	58.6%	_
Dong et al., 2017 (China)^30^	31	206	76	No	MRI	64.5%	27.18%
Verma et al., 2017 (India)^8^	104		74	Yes	MRI	71.15%	_
Wen et al., 2017 (China)^31^	28	59	33	No	CT/MRI	92.8%	11.86%
Fang et al., 2017 (China)^32^	23	77	49	No	MRI	100%	33.76%
Bojja et al., 2018 (India)^9^	123		26	Yes	CT/MRI	21%	_
Bansal et al., 2020 (India)^33^	8	-	8	No	CT/MRI	100%	-
Basavarajappa et al., 2020 (India)^10^	15	60	27	Yes	MRI	86.7%	23.3%

CT: computed tomography; MRI: magnetic resonance imaging; PRES: posterior reversible encephalopathy syndrome.

Most of the articles were published during the past decade. About one-third of the articles originated from North America and Europe, accounting for 13.6% of the cases (n = 207). Another third of the articles were from India and Pakistan, contributing to 26% (n = 400) of the cases. The remaining articles were from Turkey, China, Japan, Brazil, and Mexico, contributing 60% (n = 912) of the cases. There were no studies from low-income countries.

### Frequency of PRES

The overall prevalence of PRES with eclampsia in the included studies was 66.7% (569 PRES-diagnosed women in 852 women with eclampsia). Restricting to the six studies^5–10^ which reported neuroimaging on presumed consecutive or “all” women, the frequency was 51.4% (176/342 women).

Among the 15 studies which reported on 667 women with severe preeclampsia, PRES was present on imaging in 4–35% of cases, with an overall prevalence of 24% (161/667). Restricting to the three studies which reported neuroimaging on “all” women with severe preeclampsia,^5,7,10^ the frequency was 19.8% (24/ 121 women).

### Location of PRES lesions

All parts of the brain were reported to be involved in women diagnosed with PRES. The most frequent reported involvement of the brain was in the occipital lobe (84.2%), followed by parietal (67.9%), and frontal area (32.2%) in a total of 387 cases.^6,10,12–17,20,21,24–28,30–32^

### Presenting signs/symptoms

In the studies that provided information on symptoms^5–8,15–18,19,21–26,28–30,31–33^ of PRES, 567 women were evaluated for the presence of headaches, seizures, visual changes, and altered mental status. Seizures (77%) and headaches (70.9%) were the most common symptoms among these women. Other symptoms included visual changes (47.8%) and altered mental changes (30%).

### Blood pressure measurements

There was no significant difference in systolic or diastolic blood pressure measurements between the PRES group and the non-PRES group in women with eclampsia/severe preeclampsia.^6–8,22,28,30,32^

In only one study, Matsuda et al.^5^ reported statistical significance in differences of diastolic blood pressure measurement among women with PRES (114.0 + 2.0 mmHg) compared to those with normal MRI (102 + 2.0 mmHg) {*p* = 0.0007},while no statistical significance was observed in systolic blood pressure among the two groups.^5^ See Supplementary Table 1.

### Maternal outcomes

Seven studies^7,8,13,19,22,31,33^ reported on maternal intracranial hemorrhage with PRES. The overall reported cases of intracranial hemorrhage were 11.2% (24/214).

Fifteen studies reported on maternal mortality. The pooled mortality rate among all 15 studies was 5.3% (21 deaths in 395 PRES confirmed cases).

Eight of those studies^5,6,14,23,25,26,29,30^ reported no mortality in 204 preeclamptic/eclamptic women diagnosed with PRES. The remaining seven studies stated the following rates of maternal PRES-related death:

Fisher et al.^28^ reported a mortality rate of 1 in 9 mothers with PRES. Junewar et al.^22^ reported the deaths of 6 out of 35 women with PRES. While Verma et al.^8^ reported the highest number of maternal mortality, 9 deaths out of 74 women. Paul et al.^20^ and Striano et al.^12^ reported 2/6 women and 1/7 women, respectively. Finally, Fang et al.^32^ and Bansal et al.^33^ reported each the death of 1 woman out of 49 and 8 women with PRES, respectively.

### Delivery outcomes

There were four studies that reported on the mode of delivery of PRES women. Wen et al.^31^ reported cesarean delivery for all women with antepartum PRES (n = 15) and 81% (17/21) of patients with postpartum PRES. Demir et al.^18^ reported a cesarean rate of 55.7% in 61 PRES women. Shah et al.^14^ reported that among 40 PRES-diagnosed women, the cesarean delivery rate was 42.5%. Fang et al.^32^showed a statistically significant higher rate of cesarean delivery in women with PRES (94%) and those without PRES (76%) groups. See Supplementary Table 2.

We were unable to abstract data on fetal outcomes such as fetal growth restriction, preterm deliveries, stillbirth, and neonatal death as the data on these outcomes from the studies was limited.

## Discussion

### Main findings

The findings from our review suggest that PRES is frequently detected in women with eclampsia and with severe preeclampsia presenting with neurological symptoms. The majority of women in our analysis were from well-resourced countries (73.6%, n = 1119), with the remaining from lower-middle-income countries. There were no studies from low-income countries. This is to be expected since head imaging may not be easy to access in places with few resources.

The reported prevalence of PRES in those with eclampsia from all studies ranged from 21% to 100%, and for severe preeclampsia, 4% to 35%. The pooled prevalence from these studies was 66% in those with eclampsia and 24% in those with severe preeclampsia. When limiting our analysis to studies that included all comers, the incidence was similar at 51.4% and 19.8%, respectively, for women with eclampsia and symptomatic women with preeclampsia.

From these studies, we were unable to ascertain predictive variables associated with the development of PRES. However, it is clear that findings of PRES on neuroimaging are common in women with neurological symptoms (headaches, vision disturbance, seizures) who develop severe preeclampsia.

We found the maternal mortality rates reported with PRES in eclamptic and preeclamptic women from pooled studies to be quite high at 5.3%. This underscores the need to develop guidelines on brain imaging and delivery intervention, particularly for women with severe preeclampsia, who present with symptoms that could suggest the presence of PRES.

### Strengths and limitations

There are some limitations associated with our systematic review. Our results are derived from case series, which are usually small and subjected to selection biases. Neuroimaging was likely only performed in women with severe symptoms and in large centers and well-resourced healthcare countries with the availability of these imaging tests. Because large prospective studies in this area cannot be feasibly achieved from any one center, pooled results from smaller retrospective studies offer the best estimate of the frequency of PRES in these women.

Another limitation to our study is the use of CT scan versus MRI to diagnose PRES. Although both CT scan and MRI can be used in the diagnosis of PRES, MRI is more sensitive and specific than CT in diagnosing PRES.^34^ Most studies in our review reported the use of MRI for the diagnosis of PRES. Of the 29 included studies 16 used exclusively MRI as their imaging modality representing a total of 882 (58%) women, the remainder of the studies used both MRI and/ or CT; hence, our estimated prevalence is likely accurate.

### Interpretation

Although the exact pathophysiology of PRES is still not well understood, it is hypothesized that a disruption of the cerebral blood flow autoregulation in the brain in the form of a reduction in the cerebrovascular resistance results in hyperperfusion and blood–brain barrier disruption and vasogenic edema.^35^ In our analysis, we found that the most common neurologic symptom in preeclamptic women diagnosed with PRES was headache. More details about the nature of the headache were not available. We were similarly unable to detect any differences in BP measurements in women with and without PRES.

Our review is the first of its kind to highlight the fact that PRES is common in pregnant women with severe hypertensive disorders. Another important finding from our review is that neuroimaging for PRES demonstrated the involvement of all parts of the brain and was not limited to the posterior lobes. Finally, PRES is associated with significant maternal morbidity such as intracranial hemorrhage (11.2%) and maternal mortality (5.3%). The long-term implications following the development of PRES are unknown.

## Conclusion

PRES is a common finding in women with eclampsia and severe preeclampsia who undergo neuroimaging. Involvement of all regions of the brain is seen in these women with PRES.

The need for neuroimaging in these women, especially if they present with eclampsia or neurological symptoms requires further clarification.

### Research recommendations

Preeclampsia and eclampsia remain a leading cause of maternal mortality, particularly in developing countries.^36^ The presence of PRES in these women necessitates prompt management or delivery to prevent maternal sequelae or death. Developing clear guidelines for neuroimaging for all women diagnosed with eclamptic and especially those diagnosed with preeclamptic in whom there are neurologic manifestations is of immense importance for frontline clinicians. Long-term outcomes in these patients should also be investigated.

## Supplemental Material

sj-docx-1-obm-10.1177_1753495X221150302 - Supplemental material for A systematic review of posterior reversible encephalopathy syndrome in pregnant women with severe preeclampsia and eclampsia Click here for additional data file. Supplemental material, sj-docx-1-obm-10.1177_1753495X221150302 for A systematic review of posterior reversible encephalopathy syndrome in pregnant women with severe preeclampsia and eclampsia by Dalal A Tawati and Wee-Shian Chan in Obstetric Medicine
